# The representational dynamics of the animal appearance bias in human visual cortex are indicative of fast feedforward processing

**DOI:** 10.1162/imag_a_00006

**Published:** 2023-08-10

**Authors:** Chiu-Yueh Chen, Gaëlle Leys, Stefania Bracci, Hans Op de Beeck

**Affiliations:** KU Leuven, Leuven Brain Institute, Brain & Cognition, Leuven, Belgium; University of Trento, CiMeC Mind/Brain Institute, Rovereto, Italy

**Keywords:** animacy perception, EEG, multivariate pattern analysis, representational similarity analysis

## Abstract

The human visual system has a seemingly unique tendency to interpret zoomorphic objects as animals, not as objects. This animal appearance bias is very strong in the ventral visual pathway as measured through functional magnetic resonance imaging (fMRI), but it is absent in feedforward deep convolutional neural networks. Here we investigate how this bias emerges over time by probing its representational dynamics through multivariate electroencephalography (EEG). The initially activated representations to lookalike zoomorphic objects are very similar to the representations activated by animal pictures and very different from the neural responses to regular objects. Neural responses that reflect the true identity of the zoomorphic objects as inanimate objects are weaker and appear later, as do effects of task context. The strong early emergence of an animal appearance bias strongly supports a feedforward explanation, indicating that lack of recurrence in deep neural networks is not an explanation for their failure to show this bias.

## 1 Introduction

The sensory systems of all animals have evolved and developed to detect the most important events in their environment. In many cases, those events are related to the presence of other animals. Prey animals have to detect predators, such as shown by the special sensitivity of rodent behavior and neural responses to looming objects that might signal the approach of a predator bird (Li et al., 2021; Yilmaz & Meister, 2013). The neural systems of predators are set up to detect and catch prey, illustrated by the existence of fly detectors in the frog’s brain (Barlow, 1953) and prey-catching behavior in mice (Hoy et al., 2016). Many animals care about processing the behavior of conspecifics, resulting in elaborate processing of social stimuli (Powell et al., 2018; Sliwa & Freiwald, 2017).

In the human visual system, studies have revealed the existence of brain regions specialized for socially relevant stimuli such as faces and bodies (Downing et al., 2001; Kanwisher et al., 1997). These regions display a sensitivity for the degree of animacy, with a graded selectivity for how similar the face and body properties of a particular animal are to the human face and body (Ritchie et al., 2021). As a result, animacy comes out as a primary dimension characterizing object representations in human cortex and perception (Bracci & Op de Beeck, 2016; Kriegeskorte, Mur, Ruff, et al., 2008; Mur et al., 2013; Yargholi & Op de Beeck, 2023). In addition to this general selectivity for animacy, human visual cortex also shows a bias to process nonanimate or ambiguous stimuli as being animate. The term “pareidolia” is used for the general phenomenon of giving a meaningful interpretation to a random pattern or shape. Very often, this interpretation is in terms of an animal form or face. Examples from daily life are numerous. We see all sorts of shapes, mostly animals, in clouds. We detect faces and human forms in rock formations and pizza. In the lab, participants interpret shape stimuli as complex animate forms even when performing simple and boring discrimination tasks (e.g., Op de Beeck, 2012; Op de Beeck et al., 2003). Perceived curvature might be a particularly important mid-level perceptual feature for this perception of animate forms (Long et al., 2017).

In some cases, the illusory perception of animacy dominates the overall processing of the presented objects in visual cortical processing, resulting in an animal appearance bias. Bracci et al. (2019) introduced a stimulus design with so-called “zoomorphic” or “lookalike” objects: nonanimate objects that are made to look like an animal, such as a cow-shaped mug. It is still easy to interpret the lookalike objects for what they really are, objects, rather for what they appear to be, animals. When judging similarity, human observers considered the lookalike objects as somewhere in between animals and objects, a bit closer to inanimate objects than to animals. Feedforward deep neural networks (DNNs) exhibited the same behavior to an even greater extent, grouping the lookalike objects with inanimate objects. We could refer to this tendency as an object bias. Nevertheless, the neural response to these lookalike objects as measured through functional magnetic resonance imaging (fMRI) was almost indistinguishable from the response to actual animate objects, showing that human visual cortex is strongly affected by the appearance of the stimuli as animals. This result was even found when subjects were doing a task in which they had to group the lookalike objects with objects.

However, it is unclear how this animal appearance bias emerges during information processing. By using fMRI, Bracci et al. (2019) obtained a time-averaged view of representational similarity. Such data cannot distinguish different hypotheses about how representations evolve over time. A first possibility is that the animal appearance of the lookalike objects is detected early on in the first feedforward sweep of information processing. This would be consistent with the findings from a recent study on face pareidolia (Wardle et al., 2020). Stimuli that elicit face pareidolia are associated with an increased activity in face-selective brain regions and early face-selective electrophysiological responses (Wardle et al., 2020). Note though that despite the speed of processing of illusory faces, early electrophysiological responses to an illusory face were still more object-like than face-like, as was also the case for the (time-averaged) fMRI responses. In contrast, in Bracci et al. (2019), the lookalike objects are processed as less object-like than animal-like. This difference complicates the generalization between these two phenomena. Furthermore, the very stereotypical nature of face templates might speed up face detection relative to the detection of animal appearance, which is indeed supported by the earlier emergence of face clusters compared to animate/inanimate clusters in cortical representational spaces (Kietzmann et al., 2019). As a result of these differences, it is uncertain to which extent early feedforward processing would underlie the strong animal bias.

A second hypothesis about the emergence of the animal bias is that the processing of the animal-like appearance of lookalike objects might be present from the start but in addition increases over time. Such increase could depend on recurrent processing after the initial feedforward sweep of information processing. Recently, there have been several reports that feedforward DNNs cannot fully capture the representational dynamics in human visual cortex (Kietzmann et al., 2019) and cannot explain human performance in difficult object recognition tasks (Seijdel et al., 2021; Tang et al., 2018). The very different behavior of feedforward DNNs (lookalikes processed as objects) and human visual cortex (lookalikes processed as animals) might be due to the fact that human visual cortex relies upon recurrent processing to process the animal-like appearance of these lookalikes. The gradual increase in the animacy representation might also explain why the animal bias measured by Bracci et al. (2019) is much stronger than face pareidolia effects (Wardle et al., 2020).

In this study, we investigated the representational dynamics and task dependence of the animal bias for lookalike zoomorphic objects using electroencephalography and fMRI-EEG fusion. We find that the initially activated representations to lookalike objects are very similar to the representations activated by animal pictures. Neural responses that reflect the true identity of the lookalikes as inanimate objects are weaker and appear later. Task effects of the relevance of the animal appearance versus object identity were relatively minor and confined to later time points. In sum, the bias to process lookalike objects as if they are animals is particularly strong in the initial response to these lookalike objects.

## 2 Methods

### 2.1 Participants

A total of 30 healthy volunteers (23 females; mean age, 21 years) were recruited online through a university online recruitment system (SONA). The volunteers received either course credit or monetary rewards. Most volunteers were belonging to the student population of KU Leuven, and there was no restriction in terms of gender. This study was approved by the KU Leuven Social and Societal Ethics Committee (G-2020-2379). All participant’s data were organized according to the brain imaging data structure (BIDS) (Pernet et al., 2019).

### 2.2 Stimuli and experimental design

**Fig. 1.**
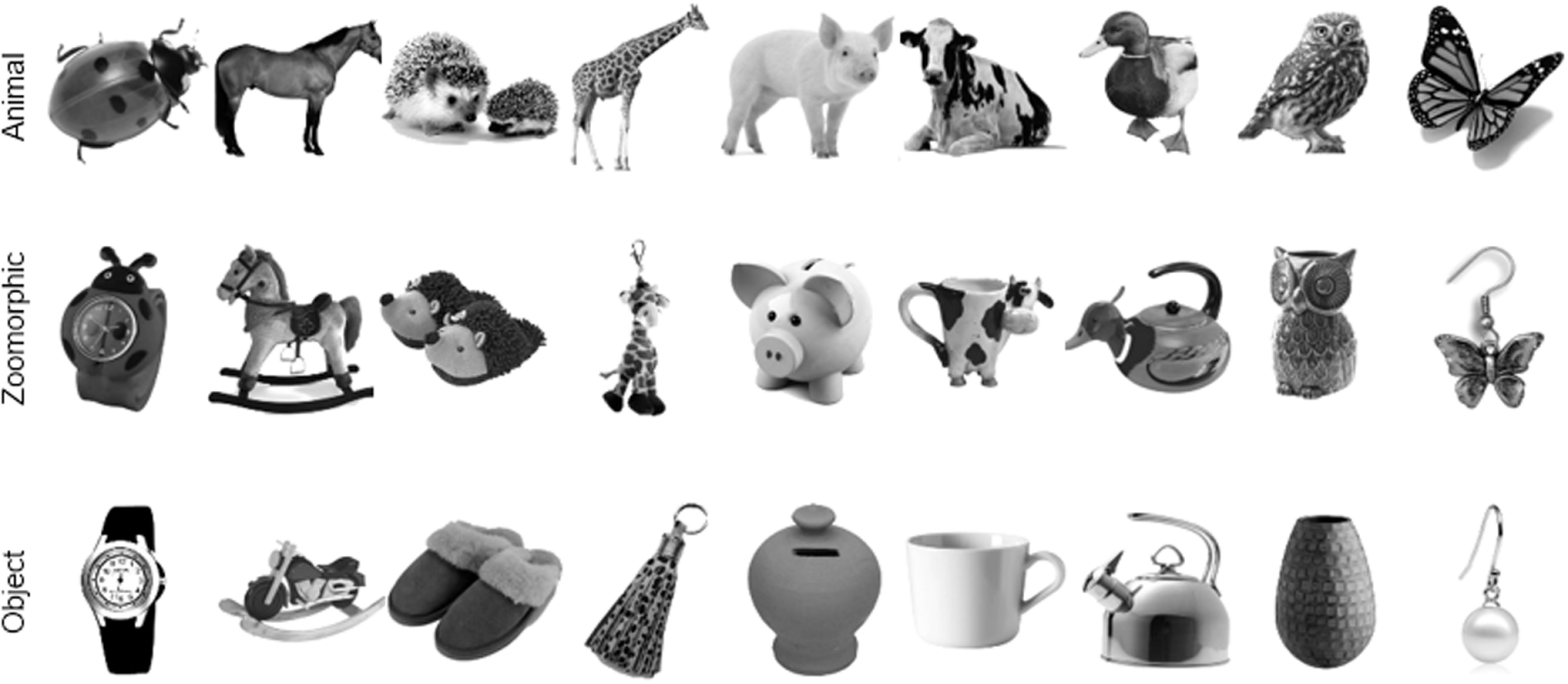
Experimental stimuli. The stimulus set consisted of 27 stimuli in three categories (animate, zoomorphic, and inanimate objects) and was used in the study of Bracci et al. (2019).

Stimuli consisted of 9 triads, resulting in a total of 27 stimuli (see Fig. 1). Within these triplets, visual appearance and category information (animacy) were manipulated. Each triplet consists of one animate, one inanimate, and one zoomorphic object that looks like the animal and is matched to the object (e.g., a cow, a mug, and a cow-shaped mug). All of the images were gray-scaled and used before by Bracci et al. (2019).

Participants performed two tasks: an animacy task and an animal appearance task. In the animacy task, participants judged animacy (“Does this image depict a living animal?”). Participants responded “no” to the objects as well as to the zoomorphic objects. In terms of response tendency for the zoomorphic objects, this can be rephrased as an object bias (lookalikes classified with the objects). In the appearance task, participants judged animal appearance (“Does this image look like an animal?”). Participants responded “yes” to the animals as well as to the zoomorphic objects, which we refer to as an animal (appearance) bias. Both task instructions were the same as used in Bracci et al. (2019).

Each task consisted of 7 runs, resulting in a total of 14 runs. During the whole experiment, the task and finger-response keys were switched every three to four runs, with the order counterbalanced between participants. The finger-response associations were altered so that one association used the left-arrow key for “yes” and the right-arrow key for “no” and vice versa in the other association.

Each run started and ended with five seconds of a fixation cross in the center of the screen, and consisted of 108 trials per run. Within each trial, an image was presented for 500 ms in the screen center followed by a blank screen for 700 ms. Participants were required to respond as accurately and quickly as possible after the stimulus presentation. No matter whether the response was made or not, a fixation cross was shown with a random intertrial interval ranging between 700 ms and 1100 ms. Responses were only recorded for the period of 700 ms after stimulus offset, which happened in 71.3% of the trials in the animacy task and 71.4% in the appearance task.

In these recorded responses, accuracy was very high for each condition in each task, with similar reaction times across conditions. In the animacy task, accuracy, and reaction time (relative to stimulus onset) were 95.4% and 699 ms for animate, 93.4% and 707 ms for lookalike, and 97.1% and 699 ms for inanimate. In the appearance task, we found 93.5% and 713 ms for animate, 91.7% and 724 ms for lookalike, and 95.8% and 710 ms for inanimate.

### 2.3 EEG recording and preprocessing

EEG signals were recorded with a 128-channel active electrode system arranged according to the extended radial system, using an ActiveTwo amplifier at a sampling rate of 1024 Hz (BioSemi, Amsterdam, Netherlands). A photosensor tracked the exact onset time of each stimulus by detecting a concurrent change from black to white in a corner of the screen.

Participants were around 60 cm away from a BenQ XL2411 screen (24 inches, 60 Hz, resolution of 1920 x 1080 pixels), in a dark room. The stimulus presentation was controlled using a script constructed with the PsychoPy experiment builder (Peirce et al., 2019). All stimuli were presented at a resolution of 324 x 324 pixels, around 5.84 degrees of visual angle.

Offline preprocessing was conducted using the FieldTrip toolbox (Oostenveld et al., 2011) in MATLAB R2020b. To reduce the slow drift noise and the power line noise, a 2 Hz high-pass filter and a 50 Hz notch filter were used. Traces were demeaned per run (baseline correction), referenced to the average of all 128 channels, and then resampled to 250 Hz.

To remove artifacts for eye movements, muscle, heartbeats, and the channels containing excessive noise, independent component analysis (ICA) was performed using EEGLAB (Delorme & Makeig, 2004). Subsequently, the components were labeled and removed using ICLabel (Pion-Tonachini et al., 2019). Afterward, the artifact-free data were segmented into 700 ms epochs from—200 ms to 500 ms relative to stimulus onset. We define stimulus onset relatively to the onset of the photosensor, which is later than the time at which the stimulus presentation script gives the command to flip the screen.

### 2.4 Category-level decoding analysis

To determine the amount of object category information contained in EEG data, decoding analyses were applied (for review, see Grootswagers et al., 2017). A temporal searchlight analysis using linear discriminant analysis (LDA) classifier was performed, as implemented in the CosMoMVPA toolbox (Oosterhof et al., 2016). We implemented this searchlight analysis nine times per participant, combining the three pairwise contrasts of the three categorical distinctions (animal, lookalike, object) with three task circumstances: both tasks together (combining data from 14 runs), and each task separately (animacy task and appearance task). The temporal searchlight analysis included the multisensor signal from all 128 sensors. The temporal neighborhood consisted of each center time point with four neighboring time points (two to either side), moving as a sliding window across all time points from—200 ms to 500 ms. The LDA classifier was trained and tested using leaving-one-run-out cross-validation.

### 2.5 Scalp topography of category-level decoding

A spatiotemporal searchlight analysis was performed with a different spatial neighborhood setting in the sensor space. For each sensor, the sensor and its nine nearest neighboring sensors in the configuration from Biosemi 128 electrode cap formed a neighborhood. After iterating across all 128 sensors and across all time points, and averaging across all participants, the resulting maps yield the topography of category-level decoding accuracy.

### 2.6 Category-level decoding across triads

We tested the generalization of category-level decoding across triads. To do this, a temporal searchlight analysis was used, now with a leave-one-triad-out cross-validation approach. A triad consists of an animate, a lookalike, and an object, shown in one column in Figure 1. In this study, there are nine triads in total. The LDA classifier was trained with all (8) triads except one, then tested to discriminate the three categories from the left-out triad. We performed this procedure 9 times, each time with another triad as the left-out triad. In this analysis, we combined both tasks together.

### 2.7 Representational similarity analysis

Representational similarity analysis was used to evaluate the similarity for all individual image pairs over time. We again used temporal searchlight analyses including multisensor patterns from all sensors and each time point plus four neighboring time points (two on each side of the center time point). For each participant and each task, the LDA classifier was trained and tested to discriminate between each pair of individual images (27 x 26 pairs), using leaving-one-run-out cross-validation. The pairwise image decoding accuracy was used as a measure of neural dissimilarity. As a result, we obtain a neural dissimilarity matrix for each time point.

The dissimilarity matrices were compared with other data modalities in the so-called “representational similarity analyses” (Kriegeskorte, Mur, & Bandettini, 2008). Only the upper half of the matrices were used in these analyses. First, the dissimilarity matrices were correlated with the predictions from two conceptual models, an animacy model and an appearance model (Bracci et al., 2019). For the animacy model, the lookalikes were expected to evoke similar activation patterns as inanimate objects, in accordance with an object bias. For the appearance model, the lookalikes were expected to evoke similar activation patterns as animate objects, showing an animal bias. Second, we performed fMRI-EEG fusion. We used the fMRI dissimilarity matrices from the three regions of interest in Bracci et al. (2019): early visual cortex (EVC), posterior ventro-temporal cortex (pVTC), and anterior ventro-temporal cortex (aVTC). The fMRI matrices are averaged across 16 participants and combined data from two tasks.

### 2.8 Statistical inference

Statistical significance was assessed using the threshold-free cluster enhancement procedure (TFCE) (Smith & Nichols, 2009) and multiple-comparison correction with null distributions created from 1000 bootstrapping iterations, all as implemented in the CoSMoMVPA toolbox. For category-level decoding and the decoding difference between category pairs, the null hypothesis of no difference was conducted by a permutation test that shuffled the category labels on each participant 100 times. For generalization across triads, the null hypothesis of chance (33.3%) was set. For individual image pair decoding, the null hypothesis of chance (50%) was set. For correlation between neural dissimilarity matrices and models and their differences, the null hypothesis of zero correlation was used. The threshold was set at *z* > 1.96 and *z* < -1.96 (i.e., TFCE corrected *p* < 0.05, two-tailed).

## 3 Results

### 3.1 How are lookalikes processed relative to animals and objects?

**Fig. 2.**
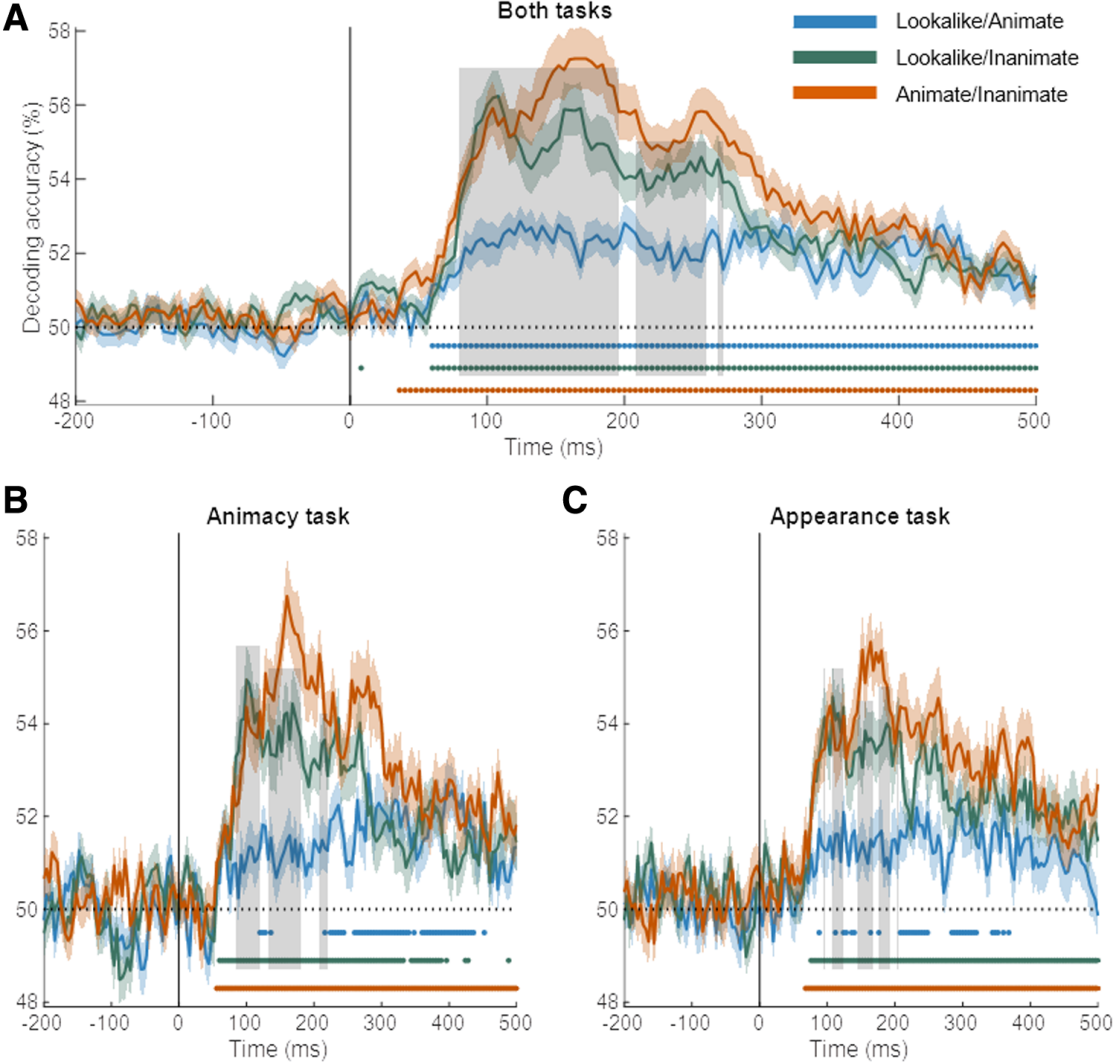
Time course of decoding for condition pairs. The conditionwise decoding accuracies over time are shown for the data combined across the two tasks (A), for the animacy task (B), and for the appearance task (C). Different lines show the decoding performance for each condition pair: animate against lookalike (blue), lookalike against inanimate (green), and animate against inanimate (orange). The light-colored regions above and below the mean lines indicate standard error across subjects (*n* = 30). Marks above the *x*-axis indicate time points where the decoding performance is significantly greater than chance. The vertical gray lines indicate the time points where the decoding difference between the lookalike and inanimate (green), and lookalike and animate (blue) is significantly different than zero.

We grouped the 27 images in three category-level conditions: Animate (animals), lookalike and inanimate (regular objects). We trained linear classifiers for the three possible pairwise contrasts between these three conditions using all trials from all runs but one, and tested these classifiers on the individual trials of the left-out run. This procedure was iterated until all runs served as left-out run. Figure 2 shows the resulting test performance averaged across all participants, taking all runs together (panel A), or separately for the two task contexts (panels B–C).

We first analyzed the data combined across the two behavioral tasks. The decoding across time for the distinction between animate and inanimate serves as a benchmark for the other distinctions (Fig. 2A, orange line). This decoding goes up toward a first peak around 104 ms, increases further to a second peak around 160 ms, and then gradually decreases toward the end of stimulus presentation but remains significant throughout.

We find almost equally high decoding for the distinction between lookalike and inanimate (Fig. 2A, green line). The initial peak around 108 ms virtually has the same height as for the distinction between animate and inanimate. Afterward decoding performance declines but remains high. In comparison, the decoding of lookalikes versus animate is much lower (Fig. 2A, blue line), significantly lower throughout most of the interval from 80 ms to 272 ms (gray area in figures). Summarized, the pairwise decoding of the three conditions suggests that lookalike objects are mostly represented as if they are animals for the first hundreds of milliseconds of the neural response.

The temporal dynamics and relative decoding strengths were very similar in the two task settings, the animacy task and the appearance task (Fig. 2B–C). In both tasks, we find a decoding of lookalike versus animate that is much lower than the decoding of lookalike versus inanimate. This is particularly striking for the animacy task. In this task, subjects are asked to group the lookalikes with the inanimate objects. Nevertheless, lookalikes are represented as most similar (lowest decoding) to the animals throughout the early part of the neural response. In later parts of the response, the decoding of lookalikes is similar for the two contrast conditions, versus animate and versus inanimate, and this is found in each task context.

**Fig. 3.**
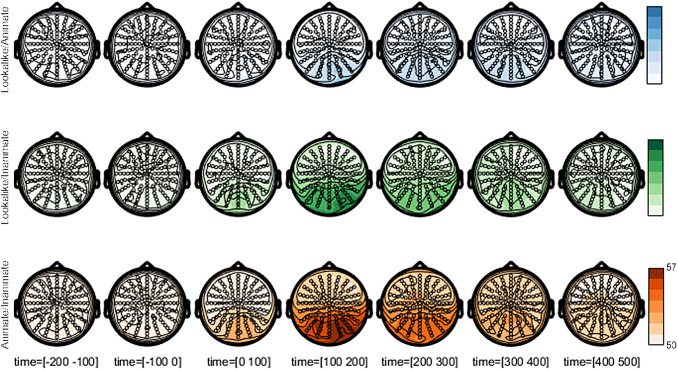
Scalp topography of decoding for condition pairs. Scalp plots show the topography of average decoding performance in seven 100 ms time intervals for each condition pair: lookalike and animate (blue), lookalike and inanimate (green), and animate and inanimate (orange).

We performed a spatiotemporal searchlight analysis to explore which electrode neighborhoods would provide the strongest signal to distinguish the three stimulus conditions in a pairwise manner. EEG is not well suited for anatomical localization, but as far as there are differences between sensors in how much they allow for category-level decoding, we would expect these sensors to be over occipitotemporal cortex where these categorical differences are typically reported in fMRI. Indeed, for each category-level distinction, we found the highest decoding around occipitotemporal electrodes, maybe with a slight bias toward the right (Fig. 3). In addition, these topographic plots further confirm the overall differences in effect size between distinctions, with larger effects for animate versus inanimate and for lookalike versus inanimate than for lookalike versus animate. However, the topographies do not suggest clear differences in the spatial distribution of the diagnostic signals between the three pairwise comparisons. Nor is there an obvious change in this topography across time beyond the expected reduction in amplitude toward later time points.

**Fig. 4.**
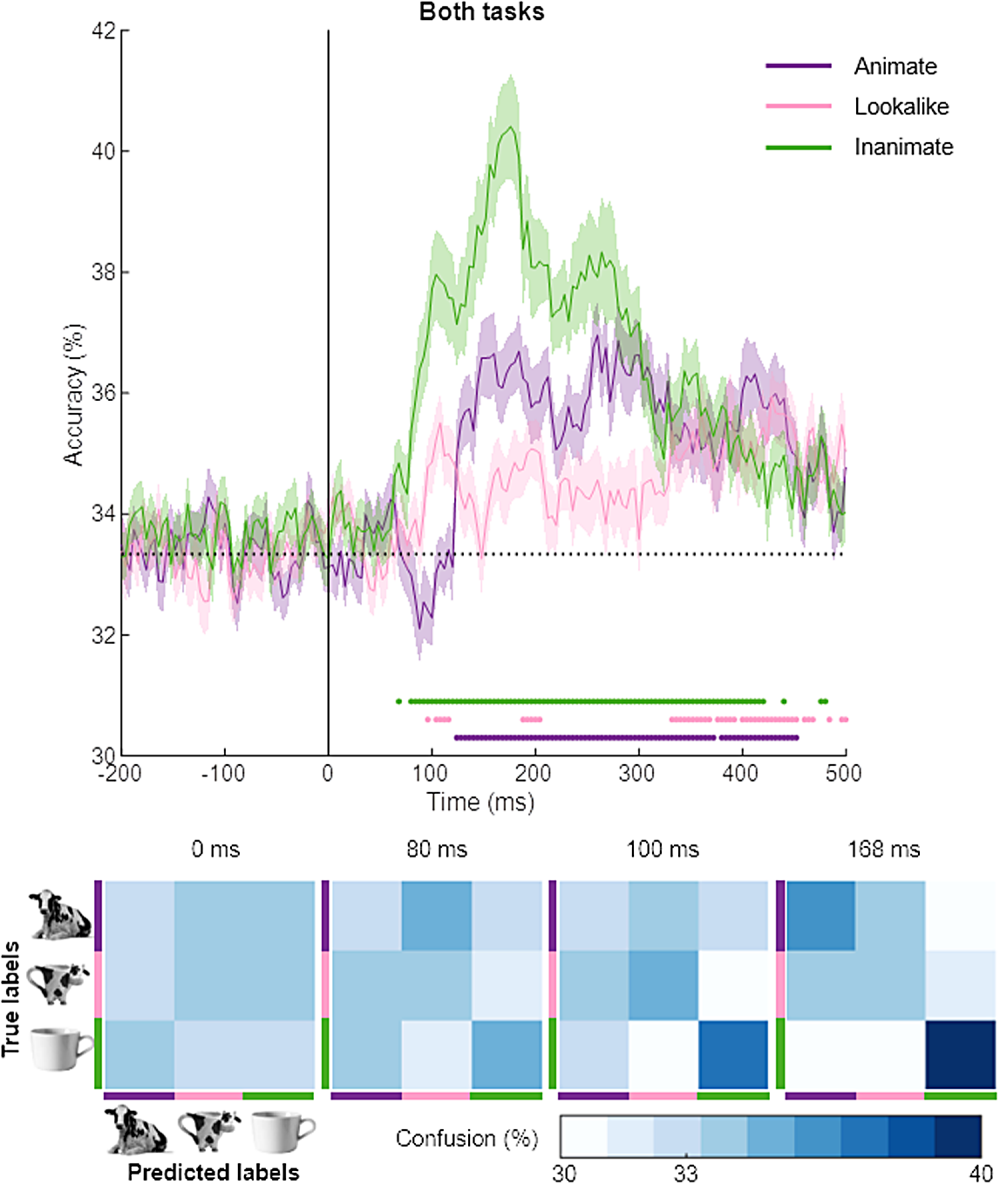
The generalization of three-way classification across triads. The time course is shown for the generalization across triads for the three-way decoding of animate, lookalike, and inanimate, computed for all trials combined across the two tasks. The top panel shows the percentage of time that the exemplar of the left-out triad of a particular category is correctly classified. The matrices at the bottom show the full pattern of confusions among the three categories. The light-colored regions above and below the mean lines indicate standard error across subjects (*n* = 30). Marks above the *x*-axis indicate time points where the decoding performance is significantly greater than chance.

To further elucidate the information contained in the decoding between the conditions of animate, lookalike, and inanimate, we performed a one-triad-out three-way decoding as also described for fMRI by Bracci et al. (2019). In this analysis, we train a linear classifier for the thee-way distinction between animate, lookalike, and inanimate using all triads but one, and test the classifier on generalization to the left-out triad. This approach guarantees that the test performance is not based on features that are specific to one or a few of the images included in the training, and would force it to rely upon more high-level object properties at the category level. The findings in Figure 4 reveal that the inanimate exemplar from the triads is classified much better than the lookalikes and animals. The 3 x 3 confusion matrices below show which pattern underlies this overall decoding difference: There is a high confusion rate between the animal and the lookalike of the triad (blue color in the corresponding off-diagonal cell), but a low confusion between lookalike and inanimate (white color). Again, the animate and lookalike are hard to distinguish, and this pattern is visible as soon as any across-triad generalization emerges in the generalization time course.

### 3.2 Representational similarity in pairwise image differences

**Fig. 5.**
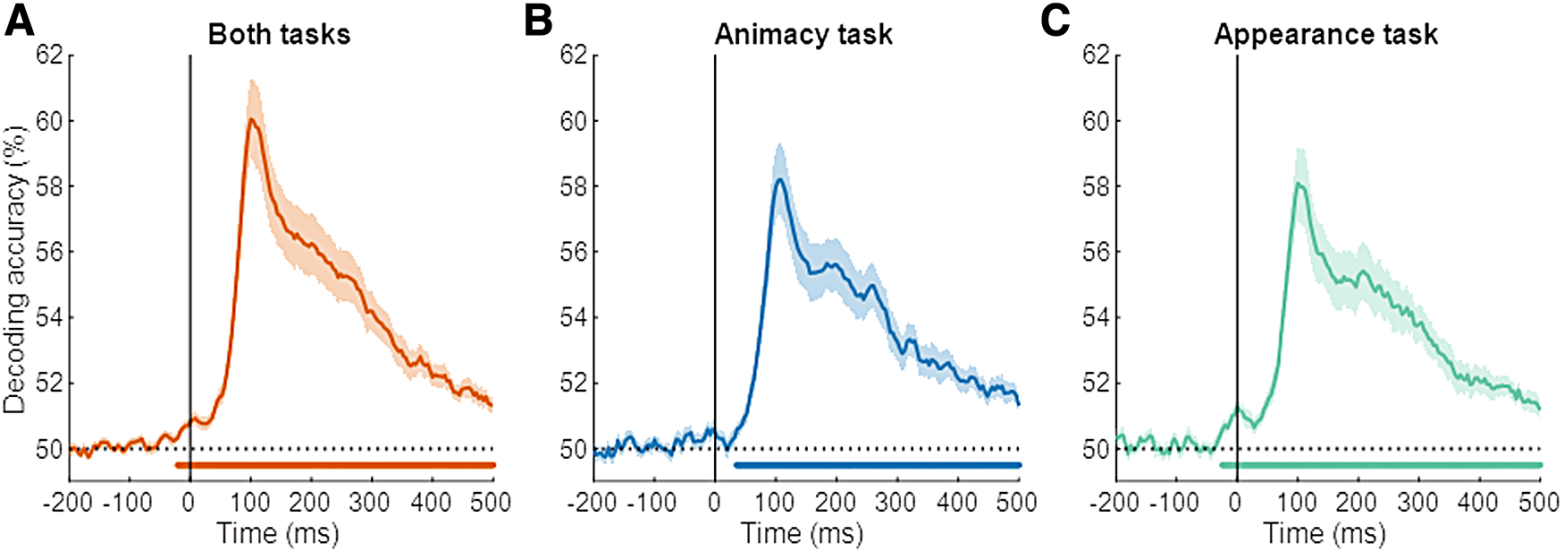
Time course of decoding for image pairs. The pairwise decoding accuracies over time, averaged across all 27 x 26 stimulus pairs, are shown for the data combined across the two tasks (A), for the animacy task (B), and for the appearance task (C). The shaded regions above and below the mean lines indicate standard error across subjects (*n* = 30). Marks above the *x*-axis indicate the time points where decoding performance is significantly different from chance.

When we train classifiers to decode the differences between the three aforementioned stimulus conditions, we lose information about differences among individual images. This grouping at the level of conditions also increases the challenge for the classification, as the classifier can only use features that are common to the images in a condition. With this in mind, it might not be a surprise that the peak decoding performance is higher when we classify individual pairs of images, despite the fact that this classification is based upon much less training data. This peak decoding of individual image classification reaches up to 60% (Fig. 5A), up from around 57% in the conditionwise decoding (Fig. 2). The curve as a function of time now shows a more prominent early peak, probably because decoding can be based upon more simple features that distinguish individual images. The curve has a very similar shape in the different task contexts (Fig. 5B–C).

**Fig. 6.**
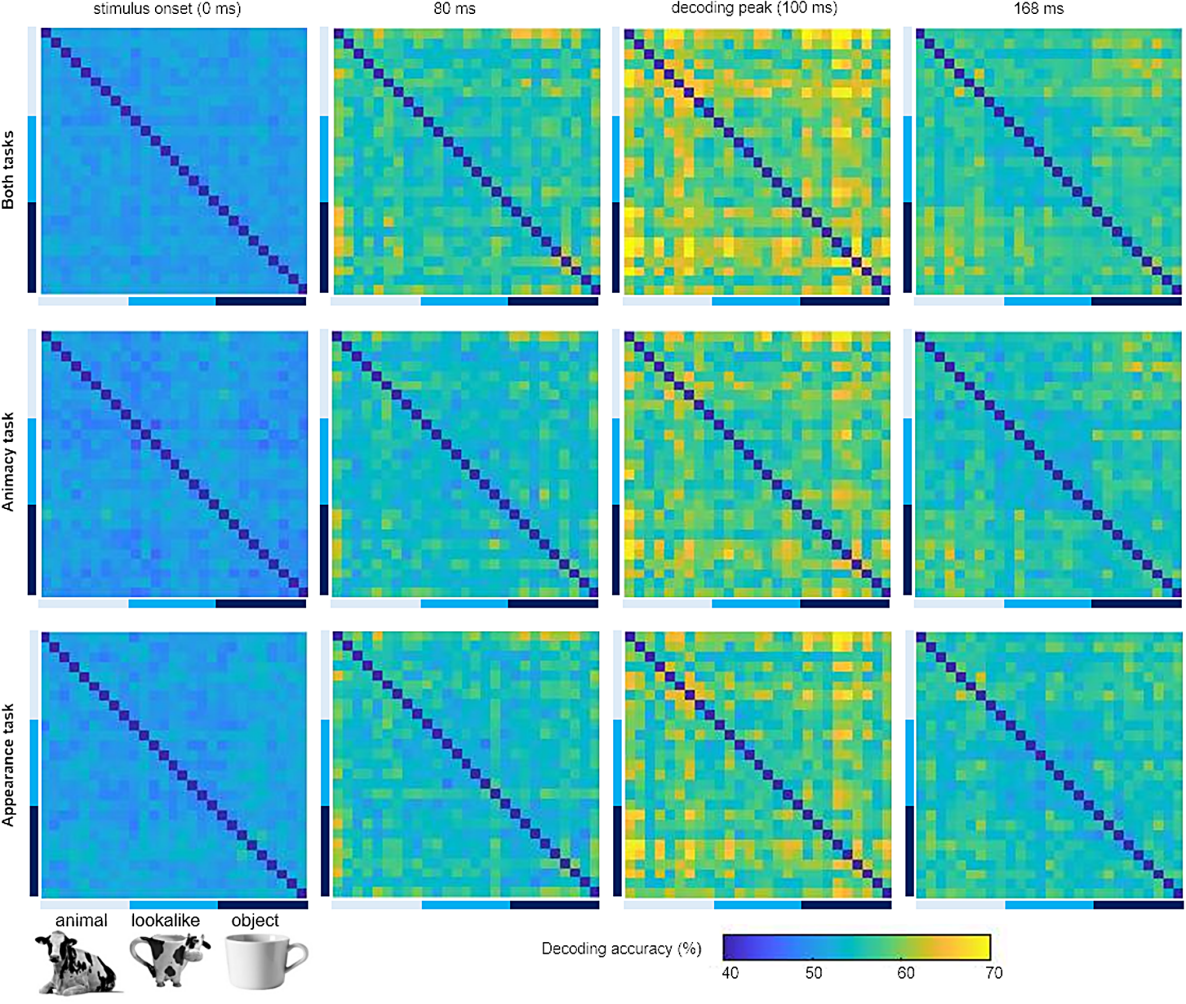
The decoding accuracies for all image pairs at 0 ms, 80 ms, 100 ms, and 168 ms. The pairwise decoding matrices at selected time points are shown for the data combined across the two tasks, for the animacy task, and for the appearance task. The higher decoding accuracy (yellow) corresponds to greater neural dissimilarity. Note that there are no data values along the diagonal, these values are pre-set at the bottom end of the color scale.

Looking beyond the overall decoding, there is a lot of systematic variation between image pairs in decoding performance. Figure 6 shows the representational dissimilarity matrices that contain the decoding of all individual image pairs. We have one such matrix for each time point. The supplemental information shows all time points as a movie. In Figure 6, we illustrate the matrices with four time points: stimulus onset at 0 ms, 80 ms, decoding peak at 100 ms, and 168 ms. We show the matrices for both tasks analyzed together and for the two tasks separately. The task-specific figures reveal the replicability of the matrices across tasks. In all tasks, we find a blue matrix (overall decoding close to chance) around 0 ms, which transforms into a green-yellow matrix around 100 ms. Around time 160 ms there is an obvious quadrant structure with a large quadrant in the top left which relates to a clustering of lookalikes with animals.

**Fig. 7.**
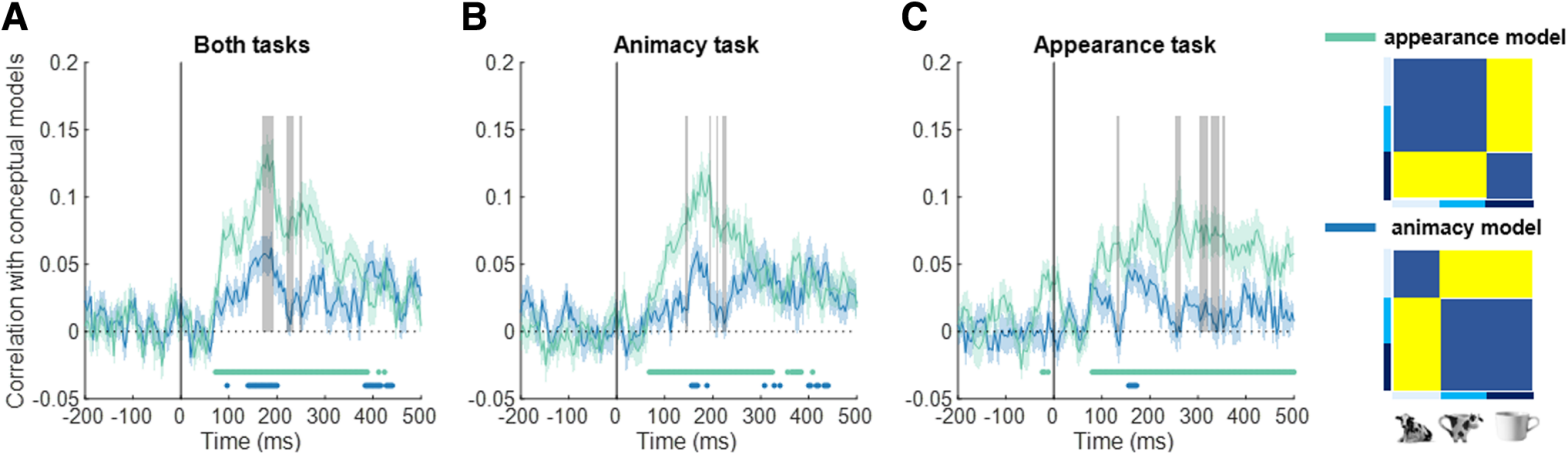
Correlation between decoding accuracy and theoretical models over time. Time course of correlation between decoding accuracy and theoretical models is shown for the data combined across the two tasks (A), for the animacy task (B), and for the appearance task (C). The shaded regions above and below the mean lines indicate standard error across subjects (*n* = 30) and the marks above the *x*-axis indicate time points where the correlation is significantly different than zero. The vertical gray lines indicate time points where the difference between the appearance model (green) and the animacy model (blue) is significantly different than zero.

To analyze this pattern more quantitatively, we correlated the matrices at each time point with the matrices from the two a priori conceptual models: the appearance model, in which lookalikes are clustered with animals, and the animacy model, in which lookalikes are clustered with the inanimate objects. Taking the data from both task settings together, we find a significant correlation with the appearance model throughout a long-time interval, but much less so with the animacy model (Fig. 7A). For part of the time, most prominently around 160–200 ms, the correlation with the appearance model is significantly higher than with the animacy model.

Through these correlations with model matrices, we also obtain a first clear effect of task setting. In the animacy task (Fig. 7B), the later part of the responses showed virtually identical correlations with the two models. In contrast, in the appearance task, there was a significantly stronger correlation with the appearance model than with the animacy model (Fig. 7C).

### 3.3 FMRI-EEG fusion

**Fig. 8.**
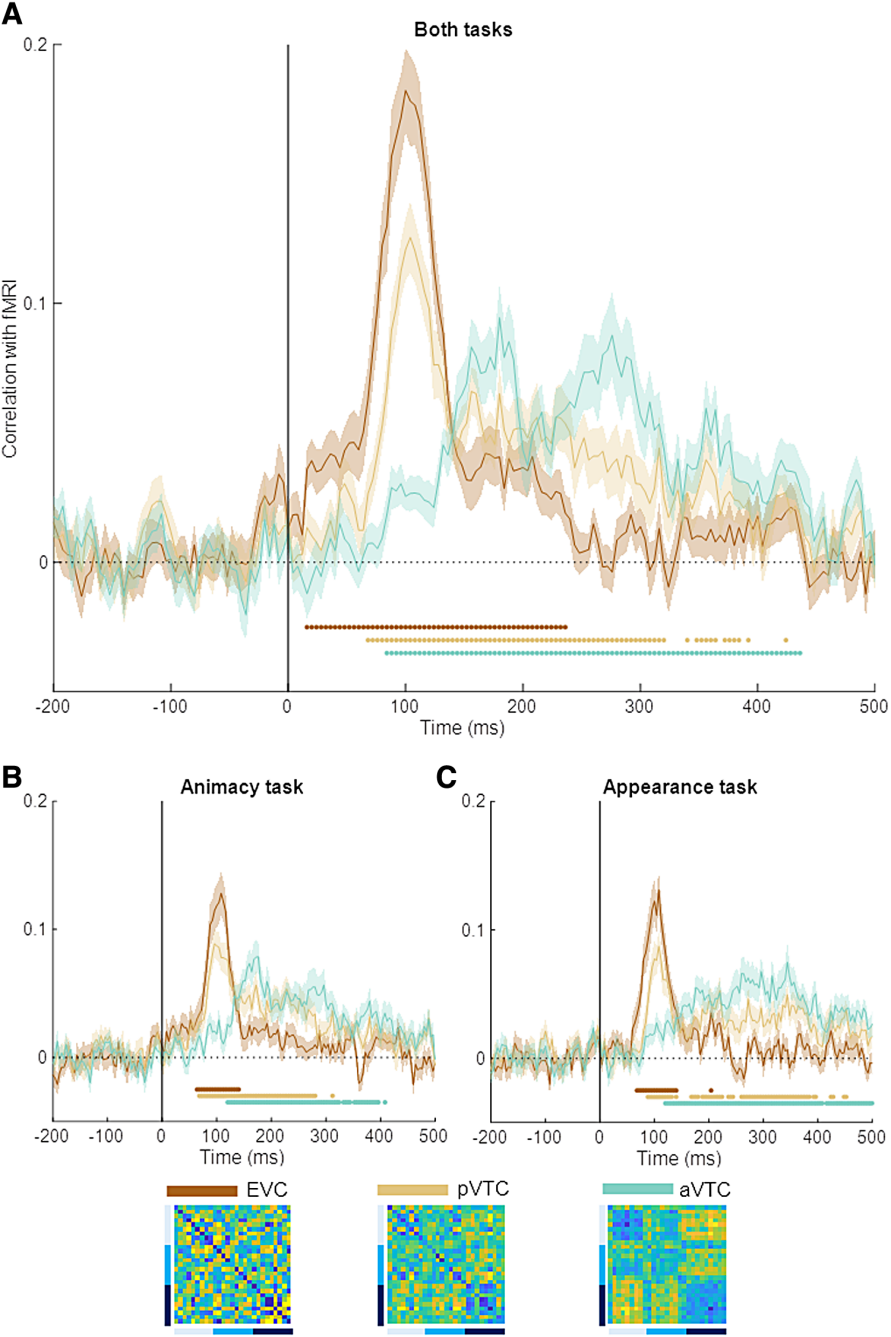
fMRI-EEG fusion. Time courses of correlation between the EEG pairwise decoding matrices and fMRI similarity patterns are shown for the data combined across the two tasks (A), for the animacy task (B), and for the appearance task (C). The shaded regions above and below the mean lines indicate standard error across subjects (*n* = 30) and the marks above the *x*-axis indicate time points where the correlation is significantly different from zero.

Bracci et al. (2019) investigated the representational similarity with the same stimulus design with fMRI. fMRI provides time-averaged data with sufficient spatial resolution to distinguish between separate brain areas. The dissimilarity matrices at the bottom of Figure 8 represent their findings for three separate ROIs: early visual cortex (EVC), posterior ventro-temporal cortex (pVTC), and anterior ventro-temporal cortex (aVTC). Bracci et al. (2019) found that the fMRI similarity patterns in EVC correlated with neither model, while pVTC and aVTC showed a significantly stronger correlation with the appearance model.

Here, we report how the time-averaged activity in these three brain regions is related to the temporal dynamics as measured through EEG (Fig. 8A). Consistent with our knowledge of the visual processing hierarchy, the representational similarity matrix in EVC correlated strongly with relatively early time points in the EEG (peak: 100 ms), pVTC still showed a clear early peak but with some sustained correlations, and aVTC correlations were initially very weak and increased toward a peak at much later time points (first peak around 156 ms). The temporal development was qualitatively similar in the two task conditions (Fig. 8B–C).

These findings reveal that the early bias to represent lookalikes as animals in EEG data cannot be due to aVTC only, as this region shows almost no correlation with EEG in these early time points. It is interesting to visually compare the EEG conditionwise decoding (Fig. 2) and representational similarity analyses (Fig. 7) with the fMRI-EEG fusion (Fig. 8). The temporal profile in the first two, summarized as a clear decoding early on but with a comparable peak later on, might be explainable by a combination of the pVTC and aVTC results in the fMRI-EEG fusion.

## 4 Discussion

We investigated the representational dynamics underlying the animal appearance bias in the visual cortical processing for zoomorphic objects. The current findings demonstrate that the animal appearance bias emerges early on in the first feedforward sweep of information processing. Early cortical responses tend to process lookalike objects more like animals than like regular, inanimate objects. A first line of evidence is the very strong early decoding of the distinction between lookalike objects and regular inanimate objects, combined with a much weaker decoding of the distinction between lookalike objects and animals. A second line of evidence is a strong early correlation with the so-called “appearance model” in which lookalike objects are clustered with animals. These effects persist for at least 200 ms, after which the animal appearance bias fades out. In the later responses, it depends a bit which task participants are performing. In particular, the stronger correlation with the appearance model persists into later time points only when participants are performing an appearance task in which they group the lookalikes with animals.

We obtained results that point toward an animal appearance bias in a very consistent way. Across both tasks together and replicated in each task context separately, we find that early responses are very much biased toward differentiating lookalike objects from inanimate objects, more so than differentiating lookalike objects from animals. The strength of this animal appearance bias, its relatively long duration, and its resilience to task effects is overall consistent with earlier findings obtained with fMRI (Bracci et al., 2019). The most important new piece of information here is that this bias is present already in the initial responses, and it is particularly strong early on. Only toward much later time points, the lookalikes seem to fall more in the middle between animals and inanimate objects. Only in these later time points, there is some indication of an effect of task context. The resilience of representational geometry to task context is consistent with previous work that showed relatively minor task effects in ventral visual cortex that increase over time, in combination with strongly task-induced representations in parietal and frontal cortex (e.g., Bracci et al., 2017; Harel et al., 2014; Hebart et al., 2018).

The spatiotemporal searchlight analyses confirm that our analyses mostly pick up selective responses in occipitotemporal regions. These analyses suggest similar topographies of the diagnostic neural signals across pairwise comparisons and across time, so we have no strong indications that different brain regions would be involved. Broadly speaking, and keeping the low spatial resolution of EEG in mind, the different categorical distinctions and the decoding at different time points seem to be supported by the same set of regions (see e.g., Graumann et al., 2022). The findings with fMRI/EEG fusion help to further specify the anatomical origin of the information in the EEG patterns. The fusion results suggest that the temporal profile of decoding and representational similarity as measured with EEG is best explained by a combination of responses from different brain regions as measured with fMRI. More specifically, the EEG time course seems best explained by a combination of posterior and anterior ventral occipitotemporal regions. This combination might also cause the double peak in object decoding that has been observed in earlier studies (e.g., Fig. 2 in Cichy et al., 2014; Figs. 3 & 5 in Mohsenzadeh et al., 2018; Fig. 2 in Robinson et al., 2019; Fig. 5 in Wardle et al., 2020).

The strong early emergence of an animal appearance bias strongly supports the first hypothesis that the processing of the animal appearance in lookalike objects is carried out in the initial feedforward sweeps of information processing. This outcome is consistent with the findings of Wardle et al. (2020) on face pareidolia, in which case the similarity of illusory faces to human faces was most apparent in the early responses. Note however that the nature of the effects is very different. In the case of Wardle et al., the object images that induced face pareidolia were still mostly processed as objects. In this study, the lookalike objects are not at all clustered with objects and are processed almost completely as animal-like. The object identity of the lookalikes is not reflected strongly in the neural responses, not in the current EEG study, and also not in the prior fMRI study, despite the strength of this object identity processing in deep neural networks. Overall, the bias to process lookalike objects as animals is surprisingly strong.

One unexpected aspect of our findings is the small but significant decoding around time 0 in the Appearance task (Fig. 5C), which is also still visible when both tasks are taken together (Fig. 5A). The design, randomization of stimulus order, and analysis stream were the same in the two tasks, thus the absence of this phenomenon in the Animacy task rules these factors out as potential explanations. We also have other unpublished data in the laboratory where the pairwise decoding time course is very similar to the Animacy task here with the same analysis stream. Further, we checked explicitly that this time zero decoding is not induced by a particular aspect of our analysis stream (e.g., cut-off of high-pass filter, ICA, temporal neighborhood used for the decoding). Given the small value of this unstable baseline and the absence of obvious explanations, we assume it is a consequence of seemingly random noise in the data. Most importantly, the early animal appearance bias is very large in comparison and is prominently found in figures in which the unstable baseline does not occur (e.g., Fig. 2). Furthermore, a conservative statistical approach that takes the decoding performance at time 0 as the reference chance performance for testing significance (instead of 50%), delays the onset of significance in Figures 5A and 5C, but does not change the main findings (e.g., in Fig. 5C the decoding would be significantly different from this new most conservative baseline from 60 ms to 360 ms after stimulus onset).

While our findings are consistent with the first hypothesis in terms of feedforward processing, they contradict an explanation in terms of recurrent processing. From this perspective, the early emergence of the animal appearance bias further deepens the mystery of why deep neural networks do not show this animal appearance bias. It was an obvious way out to point to the absence of recurrent processing in these artificial networks, but this argument is no longer valid now that we know that the animal appearance bias in human visual cortex emerges in the initial feedforward sweep of information processing. Furthermore, a gradual build-up of the animal appearance bias over time as recurrent processing proceeds was also a possible explanation for why the animal appearance bias is so much stronger compared to for example face pareidolia.

The current findings will be important for constraining further computational studies aimed at understanding why the human visual system shows such a strong animal appearance bias and why it is already so prominent in the early feedforward processing of objects. One potential avenue is the implementation of a variety of training regimes that have been shown to change information processing in feedforward deep convolutional neural networks.

## Data and code availability

Stimulus presentation code, analysis code, and data are available via https://osf.io/d5egu/.

## Author contributions

Conceptualization: Chiu-Yueh Chen, Stefania Bracci, Hans Op de Beeck. Data collection: Chiu-Yueh Chen. Methodology: Chiu-Yueh Chen, Gaëlle Leys, Hans Op de Beeck. Writing: Chiu-Yueh Chen, Hans Op de Beeck.

## Funding

This work was supported by KU Leuven infrastructure grant AKUL/19/05; the KU Leuven Research Council [grant number ZKD1090, C14/21/04]; and Fonds voor Wetenschappelijk Onderzoek FWO-Flanders [grant number G0D3322N]. C.-Y.C. was supported by a KU Leuven–Taiwan doctoral fellowship; and the 2022 National Science and Technology Council Taiwanese Overseas Pioneers Grants (TOP Grants) for PhD Candidates.

## Declaration of competing interest

The authors have declared that no competing interests exist.

## Supplementary materials

Supplementary material for this article is available with the online version here:https://doi.org/10.1162/imag_a_00006.

## Supplementary Material

Supplementary Video
